# Rehabilitation of N, N′-methylenebisacrylamide-induced DNA destruction in the testis of adult rats by adipose-derived mesenchymal stem cells and conditional medium

**DOI:** 10.1016/j.heliyon.2024.e40380

**Published:** 2024-11-14

**Authors:** Mona M. Atia, Aya Ahmed Badr EL-Deen, Hanem.S. Abdel-Tawab, Alshaimaa.A.I. Alghriany

**Affiliations:** a Laboratory of Molecular Cell Biology and Laboratory of Histology, Zoology and Entomology Department, Faculty of Science, Assiut University, 71516, Egypt; b Department of Biology, Faculty of Biotechnology, Badr University in Assiut (BUA), Egypt

**Keywords:** Acrylamide, Adipose-derived mesenchymal stem cells, Conditioned media, DNA damage, Testicular toxicity

## Abstract

Environmental pollutant acrylamide has toxic effect on human health. Numerous industries such as the paper, and cosmetics, use acrylamide in their manufacturing. In certain foods, acrylamide arises at extremely high temperatures. Mesenchymal stem cells can shield different tissues from the damaging effects of free radicals induced by acrylamide. This study aimed to compare the therapeutic efficacy against acrylamide-induced toxicity between adipose-derived mesenchymal stem cells (MSCs) and their conditioned media (CM), evaluating which is more effective. Seventy adult male rats were employed in this study, distributed among 5 groups. The control group consisted of 10 rats, while each of the other four groups comprised 15 rats. The AC group received a daily oral acrylamide (AC) dosage of 3 mg/kg. In the AC + AD-MSCs and AC + AD-MSCs CM groups, after 4 weeks of AC administration, rats were injected with 0.65 × 10^6^ AD-MSCs/0.5 ml PBS and 0.5 ml of AD-MSCs CM, respectively, via the caudal vein, and were observed for 15 days. The recovery group (Rec.), subjected to 4 weeks of AC treatment, and was allowed an additional 15 days for recuperation. The result in AC and Rec. groups revealed elevated DNA damage, P53 protein levels, apoptosis, LPO, and testosterone (free and total). In contrast, the administration of CM and the transplanting of AD-MSCs decreased the levels of these proteins. According to histological analysis, treating testicular cells with AD-MSCs mitigated histopathological lesions, fibrosis, and toxicity caused by AC. The regulation of P53, LPO protein levels, and testosterone levels, supported the function of AD-MSCs in lowering testis DNA damage and apoptosis.

## Highlights

• Low dose of acrylamide at short - term exposure, and oral intake at increased P53, LPO (oxidative stress) levels, DNA damage, histopathological change, and apoptosis in the testis of rat.
• AD-MSC transplantation has lessened the adverse effects caused by acrylamide more than AD-MSC CM.
• AD-MSCs and their conditioned medium have new therapeutic against AC toxicity.

## Graphical abstract

**Graphical abstract** shows the injection of AD-MSCs and their conditioned medium demonstrate the capability to repair the rat testis damage and apoptosis induced by AC. exposure.

**Figure:**
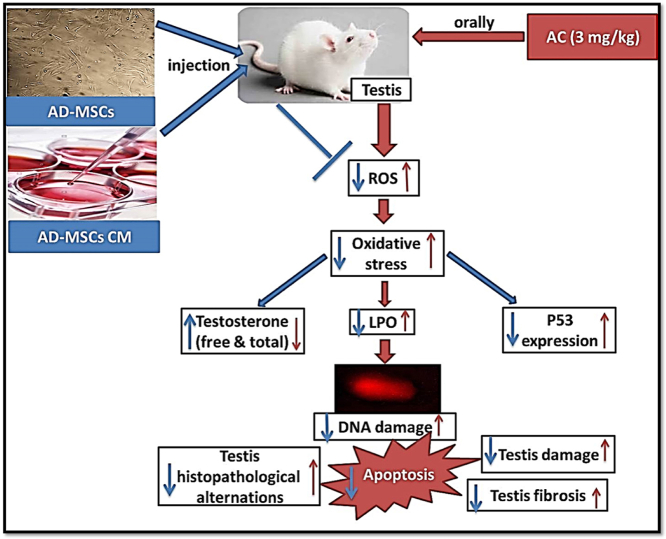

## List of abbreviation

**AC**: acrylamide
**AD-MSCs**: adipose-derived mesenchymal stem cells
**CM**: conditioned media
**AO**: Acridine orange
**P**: passage
**ROS**: reactive oxygen species
**LPO**: lipid peroxidation

## 1 Introduction

Water-soluble acrylamide (AC) is a chemical generated industrially and widely utilized in various applications, including dye synthesis, soil coagulation, wastewater treatment, paper packaging, cosmetics, and laboratory applications [1,2]. AC is formed during frying, grilling, roasting, or baking carbohydrate-rich foods, such as crackers, potato crisps, coconut, lentils, asparagus cereals, bread, and French fries at temperatures above 120 °C through interactions of amino acids (e.g., asparagine) [2,3].

Studies have demonstrated the genotoxic effects when given AC. (50 mg/kg) for 14 days to male rats [4,5]. Another study found that AC has been documented to cause genotoxic, neurotoxic, hepatotoxic, nephrotoxic, and carcinogenic effects along with developmental toxicities in mice [6,7,8]. Moreover, studies on the impact of AC on the increase of oxidative stress state, decline in enzyme activities, and inflammatory cytokine production have been reported [9], demonstrating the unavoidable adverse effects of AC on human health [2].

Elevated reactive oxygen species (ROS) levels may play an integral role in the pathogenesis of sperm chromatin/DNA damage [10]. Prolonged exposure to AC can result in apoptosis and mitochondrial dysfunction [2]. Furthermore, the AC-induced rise in p53 (tumor suppressor gene) is crucial for tumor cells' susceptibility to apoptosis expression in the liver [11]. Atia et al. [12,13] found that AC can obstruct liver detoxification, leading to degradation, necrosis, and excretion.

For this reason, many studies aim to reduce the possible side effects of ACR and some toxic agents on reproduction [14,15]. There are various studies in which mesenchymal stem cells (MSCs) have become a promising treatment for male infertility [16]. Furthermore, MSCs are immunoprivileged and genetically stable [17,18]. Adipose-derived mesenchymal stem cells (ADMSCs) are considered an attractive source of multipotent adult stem cells because they have considerable proliferative capacity, produce huge cellular numbers, and can be expanded for extended periods [19]. MSC-treated diabetic rats and spinal cord tissue damage showed decreased DNA damage as measured by the comet assay technique and histopathological improvement [20,21].

Stem cells are termed intrinsic drug stores, not only due to their differentiation capacity but also owing to their paracrine and trophic effects, serving as vehicles to deliver therapeutic agents, such as cytokines, interleukins (IL-6, IL-7, and IL-8), and apoptosis inducers. They can be genetically engineered to produce anti-tumor molecules, such as interferon β (INF β) and tumor necrosis factor-related apoptosis-inducing ligand (TRAIL) [22,23].

Numerous investigations have demonstrated that soluble mediators derived from cultivated stem cells' conditioned medium (CM) influence immunological responses via paracrine processes [24]. Many soluble mediators exist, including pro-inflammatory, pro-oxidative, vascular endothelial growth factor (VEGF), brain-derived neurotrophic factor (BDNF), hepatocyte growth factor (HGF), and transforming growth factor-beta 1 (TGF-1) [25]. Extravascular vesicles, known to have therapeutic effects in regenerative medicine, are secreted by MSCs [26,27].

The current study intended to examine the protective effects of AD-MSCs and their conditioned media against testicular damage responses in rats caused by low doses of AC at short-term exposure induced oxidative stress, inflammation, apoptosis, DNA damage, and dysfunction of the testosterone hormone. Additionally, the study compared the efficacy of adipose-derived MSCs with their conditioned media (CM) versus CM alone as therapeutic agents.

## 2 Materials and methods

### 2.1 Chemicals

Acrylamide, phosphate buffer saline (PBS), and collagenase type II were obtained from Sigma-Aldrich (St. Louis, MO, USA). Goat polyclonal IgG, mouse monoclonal IgG anti-β actin (2A3) antibody, and goat anti-mouse IgG-HRP (sc-2031) were obtained from Santa Cruz Biotechnology (Dallas, TX, USA). Gibco (Invitrogen, ca. USA) provided the RPMI-1640 (with L-glutamine) growth medium, and mouse primary anti-CD105 and CD90.1 IgG and anti-CD45 antibodies were purchased from Thermo Fisher. Ultra-Tek polyvalent goat anti-mouse HRP was obtained from Sky Tek laboratories, Logan, Utah84323, USA.

### 2.2 Ethical approval

The research methodologies employed in this study were reviewed and approved by the Faculty of Science Research Ethics Committee (FSREC) at Assiut University **(IRB no: 001-2024-0002)** in compliance with National Institutes of Health guidelines.

### 2.3 Experimental design

Seventy adult male rats were utilized in this study, divided into five groups. The control group comprised ten untreated rats (N = 10), while the other four groups included 15 rats (N = 15). The AC group rats were administered oral doses of AC (3 mg/kg) [12,13]. The AC + AD-MSCs group rats were injected with (0.65 × 10^6^) AD-MSCs/0.5 ml PBS, while the AC + AD-MSCs-CM group rats were injected with 0.5 ml of AD-MSCs CM [28,29]. at P3. The rats were injected into the caudal vein after four weeks of AC administration and left for 15 days. The recovery group rats were allowed to recover without any treatment for 15 days, and rats were narcotized by ketamine + xylazine injection (IP) and then sacrificed by slaughtering.

### 2.4 Isolation and culturing of AD-MSCs

Adult rats' visceral lipids were the source of AD-MSCs isolation. The fats were removed and sliced into 1–3 mm pieces. The adipose tissues were rinsed three times with a sterile phosphate buffer solution to extract leftover blood. 0.25 % collagenase type II in PBS with 20 % fetal bovine serum (FBS) was used to enzymatically degrade adipose tissue pieces for 45–60 min at 37 °C, shaking every 15 min. Collagenase activity was inhibited by the addition of 5 ml of FBS. After centrifugation, the pellets were suspended in 12 ml of Dulbecco's modified Eagle's medium (DMEM) culture media after removing the supernatant following a 10-min, 1800 rpm, then filtered using a 40 μm cell strainer. After that, the cell suspension was put in a culture flask at 37 °C with 5 % CO_2_ in the incubator. The cells were cultivated for about 15 days or about 80 % confluency, and AD-MSCs at passages (P3) were appropriate for transplantation [30].

### 2.5 AD-MSCs CM isolation

MSCs were cultivated in DMEM for 24 h without the addition of FBS; this allowed for the preparation of the conditioned medium. Following this time frame, the material was gathered, centrifuged for 5 min at 1000 revolutions per minute (rpm), filtered through a 0.45 μm filter to exclude cells, and stored at −80 °C until needed [28,29].

### 2.6 Immunocytochemistry investigation

Cells were fixed with 4 % paraformaldehyde for 20 min at room temperature. Three times, for 5 min apiece, cells were rinsed in PBS. Cells were permeabilized for 5 min using fresh 0.2 % Triton X-100 in PBS. Subsequently, the cells were rinsed three times in PBS for 5 min each [31]. By the manufacturer's instructions, a secondary anti-polyvalent stain was applied. The slides were incubated in a blocking buffer for 10 min. After primary antibodies CD105, CD90 (2:100), and CD45 (1:100) were incubated (1 h at room temperature), the slides were cleaned 4 times for 5 min each using PBS buffer. After applying Ultra-Tek anti-polyvalent stain and letting it sit at room temperature for 10 min, it was rinsed four times. After adding DAB chromogen to the DAB substrate mixture, the slides were quickly cover-slipped and counter-stained.

### 2.7 Comet assay investigation

Using 600 μl of low-melting agarose (0.8 % in PBS) was combined with 100 μl cell suspension for slides that had been coated. The coated slides were submerged for 15 min in a lysis buffer (0.045 M Tris/Borate/EDTA buffer (TBE), pH = 8.4, with 2.5 % sodium dodecyl sulfate (SDS). The slides were put in an electrophoresis chamber without SDS but with a TBE buffer. The settings for the electrophoresis were 100 mA for 2 min at 2 V/cm. The procedure involved staining with 20 μg/ml of ethidium bromide (EtBr) at 4 °C. Observations of EtBr-stained DNA for visualizing DNA damage were conducted using a 40× objective on a fluorescent microscope (with an excitation filter of 420–490 nm) [32]. By evaluating calculated the qualitative and quantitative levels of the tail length, tail moment, olive tail, and percentage of DNA head in five images by the open comet software.

### 2.8 Immunohistochemistry study

Tissues embedded in paraffin were removed using xylene, and rehydrated using a range of ethanol solutions. The slides were boiled in 1 mM EDTA to extract the antigens, followed by 10 min of section development in 3 % H_2_O_2_, 5 min of washing with wash buffer (1× PBS), and 1 h of room temperature blocking. Then, P53 primary antibody (1:1000) was added. After removing the antibody solution, the sections were given a 10-min wash with a wash buffer. After incubating each part for 30 min, secondary antibodies (1:5000) were added and withdrawn. After cleaning the sections, they were stained for two to 3 min with 3,3′-diaminobenzidine (DAB) and counter - stain [33].

### 2.9 Acridine orange staining for detection of apoptosis

Acridine orange (AO), in its monomeric form, exhibits metachromatic characteristics and demonstrates green fluorescence under a microscope at excitation/emission around 488 nm when exposed to blue light. Upon binding to DNA, it produces green fluorescence [34,35]. The sample was rapidly passed through 80–70–50 % alcohol, followed by distilled water, then stained with 0.01 % AO (Stock solution: 0.1 % AO in distilled water, diluted with a phosphate buffer to achieve a staining solution pH of 7.2). The sample was transferred into PBS for 1 min, differentiated for 2 min in 0.10 M CaCl_2_, quickly rinsed with a phosphate buffer, and mounted moist with a cover glass for examination [34,35].

### 2.10 Testosterone measurement

Enzyme-linked immunosorbent assay (ELISA) was used to measure free and total testosterone levels in serum samples. The methodology used was based on the methods of **Chen et al.** [36] for free testosterone and **Kovacs & Orth** [37] for total testosterone.

### 2.11 LPO as an oxidative stress marker

According to Ohkawa et al. [38], LPO in the testis was measured using malondialdehyde as thiobarbituric acid. Following homogenization, 1 % v/v DMSO was added to stop further oxidation. 0.2 ml aliquots of tissue homogenates were mixed with the reaction buffer and measured using spectrophotometry.

### 2.12 Detection of AD- MSCs in the testicular tissues

Prussian blue stain was used to highlight mesenchymal therapeutic stem cells labeled with iron oxide within testicular tissue. 50 μm iron oxide was added to 4 ml of RPMI medium and left for 30 min. Then, the mixture was centrifuged at 2000 rpm for 10 min [39]. MSCs labeled with Feridex were trypsinized, rinsed in PBS, and then reconstituted in 0.01 M PBS at 1 × 1,000,000 cells/ml concentration. The 4 μm testis sections underwent deparaffinization and dehydration, two PBS washes, and an incubation period with shaking for staining. After washing, the testicular sections were incubated for 15 min at room temperature with Perls' reagent (20 % potassium ferrocyanide and 20 % hydrochloric acid). The two solutions were combined and shaken. Sections were then rinsed in water, counter-stained with eosin, absolute ethanol, and ethanol (90–70 %), and then DPX as mounting media [40,41].

### 2.13 Histological examination

Testes were fast-fixed for histological and histochemical analysis in 10 % neutral buffered formalin (pH = 7.2). Testis sections were generated using the paraffin-embedded technique. After that, they are cleansed in xylene, and dried in ethanol solutions (ranging from 70 % to 100 %). Sections of paraffin blocks, 5 μm thick, were cut by microtome. Lastly, the standard staining protocol was carried out, using Masson's trichrome stain and hematoxylin and eosin stain [42]. On each slide stained with H & E, the thickness of the epithelium of ten seminiferous tubules was measured in micrometers, starting from the basement membrane and ending at the lumen. Leydig cells were counted in 20 randomly selected interstitial areas (a gap between three seminiferous tubules) stained with H & E.

### 2.14 The histopathological lesions examination

Ten testicular tissue lesions were evaluated by histopathological assessment, including edema, tubular atrophy, disorganized germinal epithelium, vacuolar degeneration in germinal epithelium, undefined cells of germinal epithelium, mitotic figures in primary spermatocytes, seminiferous tubules, lumen without spermatozoa, vascular congestion, vascular dilatation, and Ledig cells degeneration. The findings were categorized into four grades: (-) absent lesion, **(+)** slight (< 25 %), **(++)** moderate (from 25 to 50 %), and **(+++)** severe (> 50 %) [43]. Examination and photography were conducted by utilizing a digital camera (ToupTek ToupView, copyright© 2019, version: x86, compatibility: Windows XP/Vista/7/8/10, China), and a light microscope (Olympus CX31, Japan).

### 2.15 Statistical analysis

The values were given as mean ± SE. Student's t-test was utilized to compare parameters between two groups, and a one-way analysis of variance (ANOVA) was employed for multiple comparisons, conducted in at least three independent determinations. Analysis of variance was used for the statistical analyses, and a difference was considered significant at P < 0.001 and P < 0.05. Data analysis was performed using Prism software versions 8.4.3 (686), Fiji/Image J, and Open Comet for graph images.

## 3 Results

### 3.1 Characterization of AD-MSCs

**Fig. 1:**
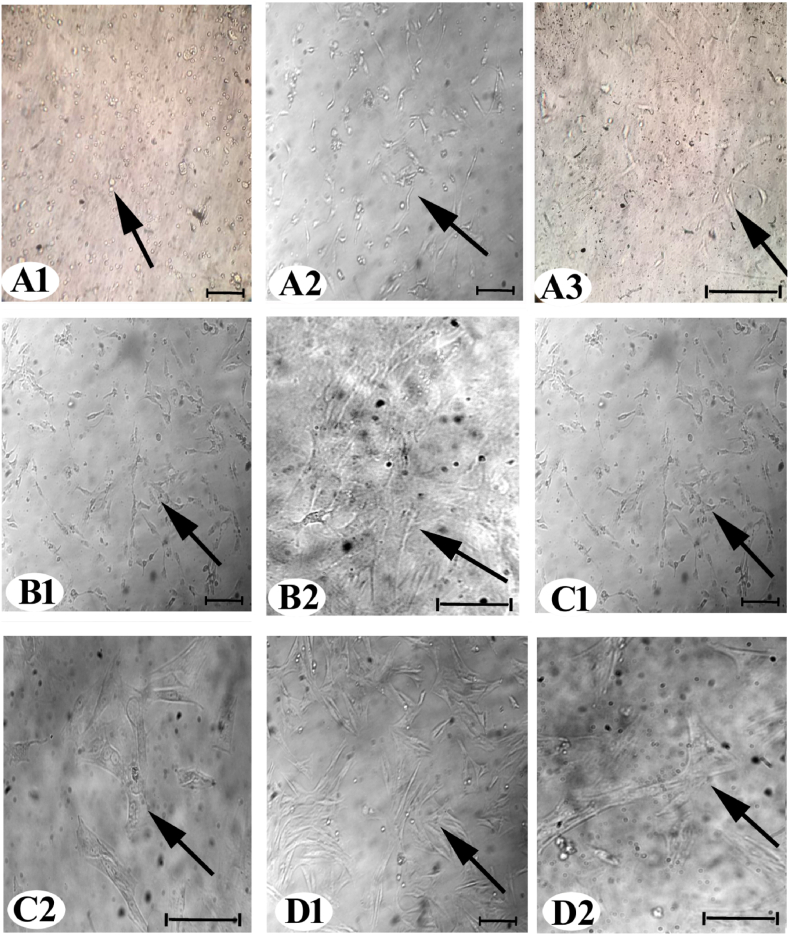
By using inverted microscopy, the morphology of AD-MSCs was observed on various days and passages. (A1) Day 0, (A2 and A3) day 2, P0, (B1 and B2) day 7, P1, (C1 and C2) day 10, P2, (D1 and D2) day 14, P3. The arrows indicate of AD-MSCs appearance (200 & 400 ×).

**Fig. 2:**
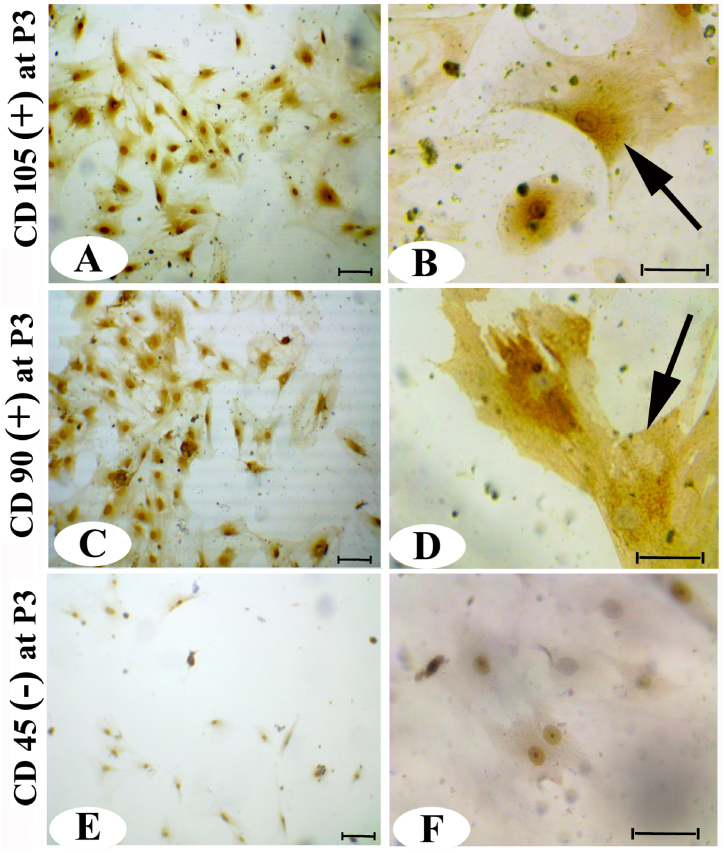
Immunocytochemistry staining of AD-MSCs at P3 showing; (A and B) CD105 and (C and D) CD90 appeared as brown dots (+) reaction (arrow), and (E and F) CD45 was used as a (-) reaction no brown dots appeared (200 × and 400 × ). (For interpretation of the references to color in this figure legend, the reader is referred to the Web version of this article.)

On the initial day of cultivation, AD-MSCs exhibited a rounded and suspended morphology (Fig. 1A1). After two days of development, the cells transitioned to thin, interconnected spindles (Fig. 1A2 and A3). In passage 1 (P1), at seven days, some AD-MSCs displayed spindly morphology (Fig. 1B1 and B2). In passage 2 (P2), at ten days, certain cells formed microscopic colonies (Fig. 1C1 and C2). By passage 3 (P3), at 14 days, some cells developed fibroblastic characteristics (Fig. 1D1 and D2). Immunocytochemistry results of AD-MSCs at P3 for CD105 (Fig. 2A and B) and CD90 (Fig. 2C and D) demonstrated positive localization in the AD-MSC cytoplasm, while CD45 exhibited a negative reaction (Fig. 2E and F).

### 3.2 DNA damage examination using comet assay

**Fig. 3:**
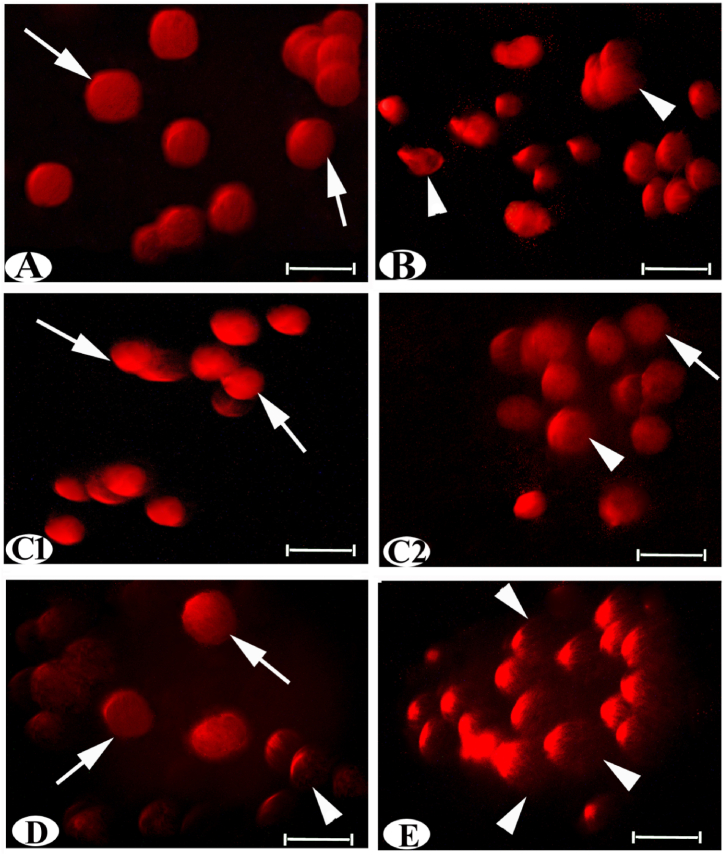
Photomicrographs of fluorescence microscope images of testis rat by Comet assay showing (A) normal testis with intact DNA, (arrow) (B) testis of AC intoxication with a high amount of damaged DNA (arrowhead), (C1, C2 and D) testis of treatment of AD-MSCs or AD-MSCs CM with a low amount of damaged DNA and intact DNA, (E) testis of Rec. intoxication with sever degree of DNA damage. Scale bar: 50 μm.

Exposure to AC for 30 days, followed by a 15-day recovery period, resulted in significant elevation of DNA damage in testis tissue. This was evidenced by a significant increase in the mean values of tail olive moment, tail moment, %DNA tail, and tail length compared to the control group. Conversely, treatment with AD-MSCs and AD-MSCs CM indicated significantly reduced DNA damage in the testis tissues, as showed by a significant decrease in the mean values of the previous parameter compared to the AC group (Fig. 3 and Table 1).

**Table (1):** The level of DNA damage assessed by comet assay in the testis of male rats at the experimental groups.

Experimental groups	Olive tail moment		Tail moment		Tail DNA %		Tail lenght	
	Mean ± SE	% of change	Mean& ± SE	% of change	Mean ± SE	% of change	Mean& ± SE	% of change
control	**4.554 ± 0.7330 a**		**0.9860 ± 0.4951 a**		**6.656 ± 2.26 a**		**11.75 ± 4.008 a**	
Ac	**61.23 ± 2.770 b**	**1244.53 %**	**88.21 ± 14.00 b**	**8846.24 %**	**55.56 ± 9.589 b**	**734.73 %**	**184.5 ± 27.38 b**	**1470.21 %**
AC + AD-MSCs	**13.01 ± 3.070 a**	**78.75 %**	**10.57 ± 5.302 a**	**88.01 %**	**23.51 ± 7.071 a**	**57.68 %**	**38.50** **± 9.509 ac**	**79.13 %**
AC + AD-MSCs CM	**27.56 ± 2.066 c**	**54.98 %**	**52.68 ± 4.232 b**	**40.27 %**	**40.75 ± 5.739 b**	**26.65 %**	**94.00 ±** **2.345 c**	**49.05 %**
Rec.	**68.61 ± 5.710 b**	**1406.58 %**	**157.4 ± 21.35 c**	**15863.48 %**	**80.94 ± 7.366 c**	**1116.04 %**	**200.8 ±** **31.45 b**	**1608.93 %**

Data are representing as the mean ± S.E. Unlike superscript letters in the same column are significantly different at P < 0.001.

### 3.3 Immunohistochemistry examination

**Fig. 4:**
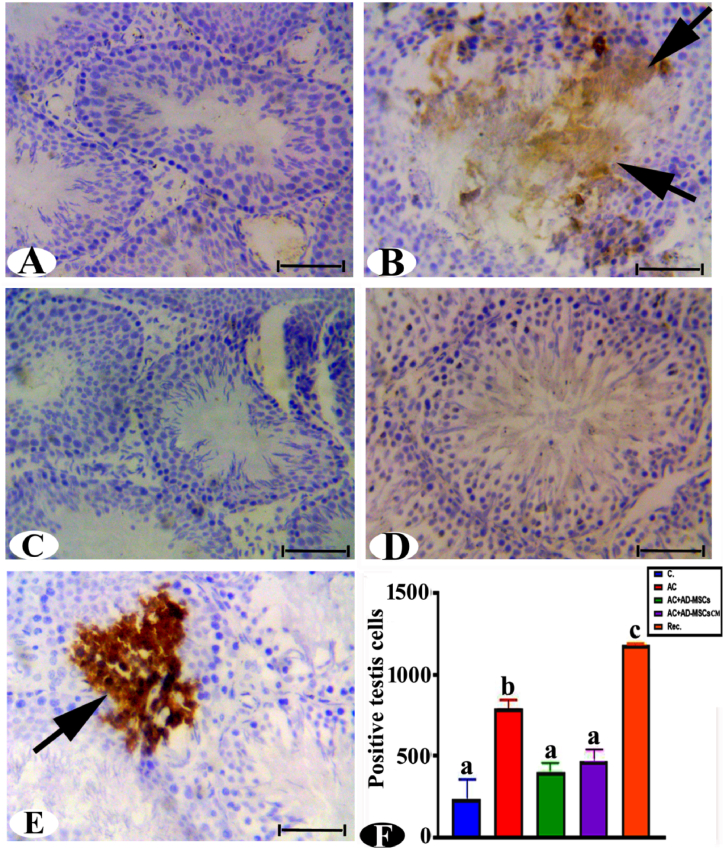
Immunohistochemistry photomicrograph examination of testis sections of rats stained with pro-apoptosis effector P53. (A) control group showing mild positive reaction P53 protein level, (B) Ac treated group showed brown immunoreactive staining of P53 with brown patches, (arrows, positive reaction), (C and D) The AC + AD-MSCs or Ac + AD-MSCs CM groups showed negative or moderate immunoreactivity of testis cells. (E) The Rec. group showed severe brown patches as positive immunoreactive staining of the testis (arrows). (F) Statistically, the values in the column with unlike superscript letters were significantly different (p < 0.001), Scale bar: 50 μm. (For interpretation of the references to color in this figure legend, the reader is referred to the Web version of this article.)

Immunohistochemical detection exhibited a negative reaction of p53 in seminiferous tubules in the control male rats testis (Fig. 4A). P53 demonstrated significantly elevated apoptotic activity, manifested by a sharp increase and large homogeneous brown patches in the spermatogonia, primary spermatocytes, and a substantial area of the seminiferous tubule in both the AC and recovery groups (Fig. 4B and E). Statistically, the levels of P53 were significantly increased (238.03 % and 405.35 %) respectively, in the previous groups compared with the control group (Fig. 4F). Conversely, P53 expression was diminished (absence of brown patches or only a few) in both AD-MSCs and AD-MSCs CM groups, indicating a reduction in P53 levels (48.76 % and 65.11 %), respectively, compared to the AC intoxication group (Fig. 4C and D).

### 3.4 Apoptosis assay by AO fluorescent staining

**Fig. 5:**
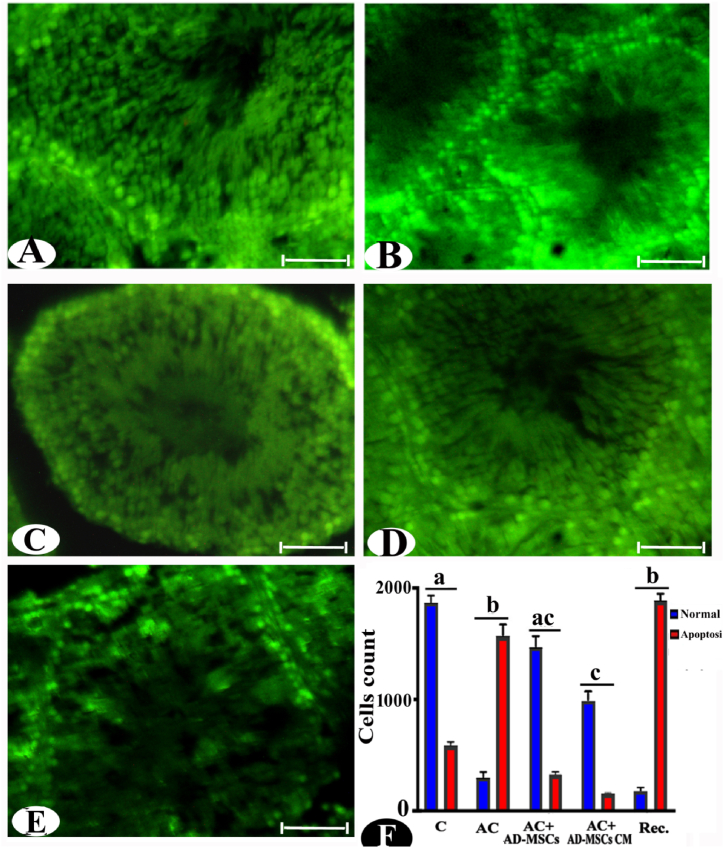
Photomicrographs of fluorescence microscope images of the experimental groups (A) control; (B) AC; (C), Ac + AD-MSCs; (D) Ac + AD-MSCs CM; (E) Rec. stained with AO fluorescent dyes. (F) Statistically, the values in the column with unlike superscript letters were significantly different. (p < 0.001), Scale bar: 50 μm.

The control group exhibited minimal cell deaths and numerous normal cells, as indicated by the presence of fully green and homogeneous nuclei, respectively (Fig. 5A and F). The effects of AC exposure followed by a recovery period demonstrated significant decreases in normal cells and increases in apoptosis, amounting to 84.41 % and 166.80 % for the AC group and 90.42 % and 220.28 % for the recovery group, respectively, compared to the control. Morphonuclear changes observed included pyknotic nuclei with dense, brightly green chromatin colors, nucleus destruction (ND), and nuclear fading (NF), indicating late apoptosis in both the AC and recovery groups (Fig. 5B, E, and F). The response of AC and the effects of AD-MSCs and AD-MSCs CM after 15 days of exposure were assessed, as depicted in Fig. 5C, D, and F. Notably, there were significant increases and decreases in normal and apoptotic cells regarding AD-MSCs (404.48 % and 79.36 %) and AD-MSCs CM (90.36 % and 239.61 %) groups, compared to the AC group. Cells stained with AO exhibited a faint green or less condensed green fluorescence corresponding to the living nuclei of normal cells.

### 3.5 Analysis of hormones

**Fig. 6:**
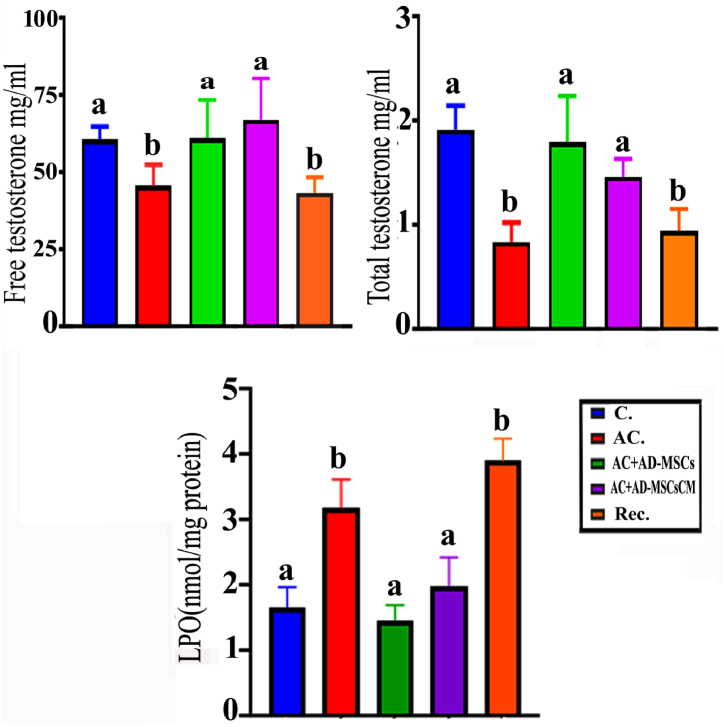
(A, B, and C) Showing the effect of AC and co-administration with AD-MSCs and AD-MSCs CM on the levels of testosterone-free & total hormones and LPO as marker oxidative stress in different experimental groups. Data are represented as the mean ± S.E. Unlike superscript letters in the same column are significantly different at P < 0.05.

The effects of AC exposure followed by a recovery period on testis induced a statistically significant decrease in the levels of free (25.00 % and 56.39 %) and total (28.67 % and 50.61 %) testosterone, respectively, compared to the control group. The response of the AC and the recovery groups to AD-MSCs and AD-MSCs CM after 15 days of exposure resulted in a significant increase in the levels of free (34.31 % and 114.89 %) and total (47.42 % and 75.27 %) testosterone, respectively, compared to the control group (Fig. 6A and B).

### 3.6 Testicular oxidative stress (estimation of lipid peroxidation)

Oral supplementation of male rats with 3 mg/kg doses of AC for 30 days, followed by a 15-day recovery, led to a significant increase (99.3 % and 125.06 %) in the level of LPO compared to control rats. Additionally, the AC + AD-MSCs and AC + AD-MSCs-CM groups exhibited a significant decrease (55.93 % and 40.04 %) in the level of LPO compared to the AC group (Fig. 6C).

### 3.7 Prussian blue staining results for stem cell homing

**Fig. 7:**
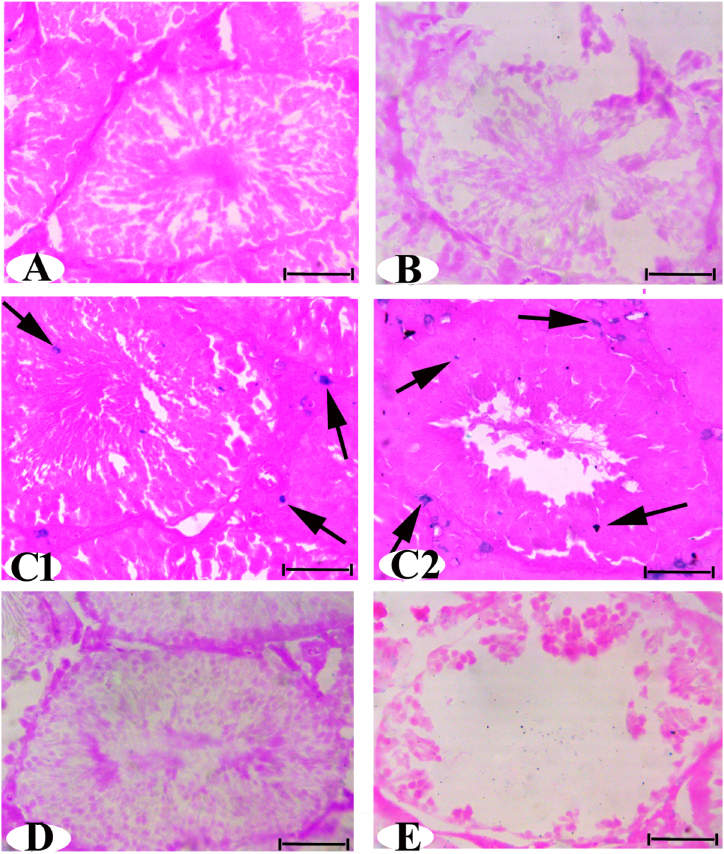
**(A, B, D, and E)** Photomicrograph with Prussian blue staining of the testes showing cells in Control, AC, AC + AD-MSCs CM, and Rec. were negative-stained cells. **(C1 and C2)** AC + AD-MSCs treated group rats showing positive-stained (arrows). (PB stain, bar = 50 μm). (For interpretation of the references to color in this figure legend, the reader is referred to the Web version of this article.)

In the testes' sections of the experimental groups control, AC, AC + AD-MSCs-CM, and recovery, Prussian blue staining revealed negative-stained cells (Fig. 7A, B, C, and E). In contrast, the AC + AD-MSCs group exhibited spindle-shaped, branched, and globular-shaped (positively stained cells) between spermatogenic cell layers and inside the lumen of the seminiferous tubules (Fig. 7C1 and C2).

### 3.8 Histopathological examination

**Fig. 8:**
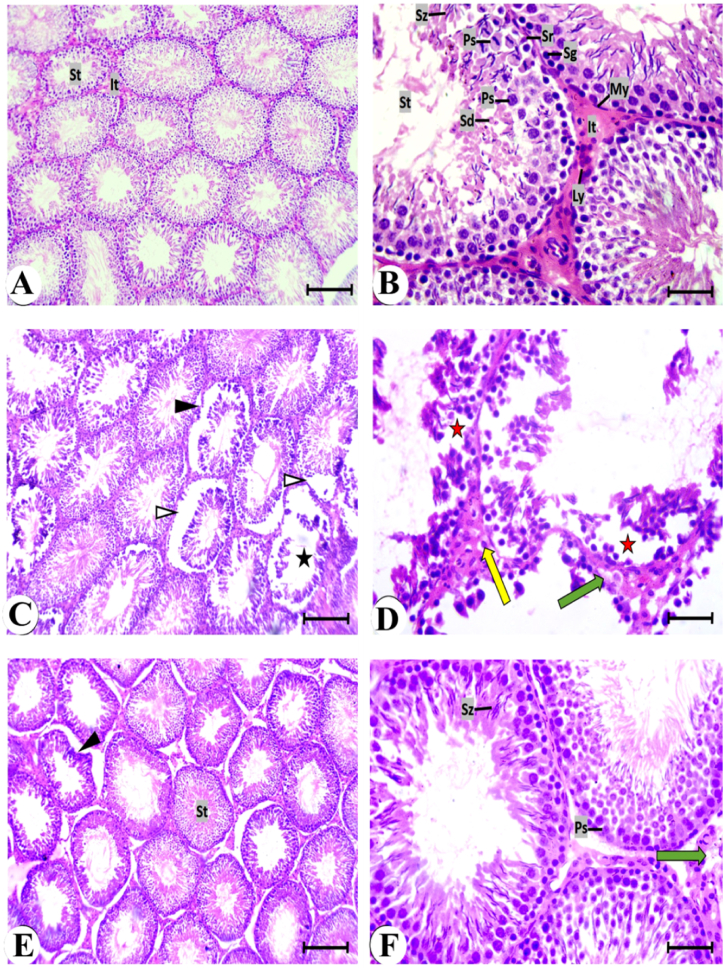
Photomicrographs of testis sections stained by H&E, bars = 100 μm (A,C and E) and 50 μm (B,D and F). **A and B:** Control group showing closely packed seminiferous tubules (St), normal interstitial tissue (It) contains Leydig cells (Ly), Myoid cells (My) and Sertoli cells (Sr). Different stages of spermatogenesis as: a spermatogonia (Sg); primary spermatocytes (Ps); spermatids (Sd); and spermatozoa (Sz). **C and D:** AC group showing irregular seminiferous tubule (▲), desquamation of the germinal epithelium to the lumen of the tubule (Δ), wide lumen devoted from spermatozoa (asterisk), spaces between ill-defined germinal cells (red asterisk), vacuolated interstitial tissue (green arrow), and Leydig cells with dense nuclei and vacuolated cytoplasm (yellow arrow). **E and F:** AC + ADMSCs group showing nearly normal seminiferous tubule (St), irregular borders of few seminiferous tubules (▲), germinal epithelium with prominent spermatocytes (Ps) and spermatozoa (Sz), and vacuolation of interstitial tissue (green arrow). (For interpretation of the references to color in this figure legend, the reader is referred to the Web version of this article.)

Sections of the testes from the control group, stained with hematoxylin and eosin, revealed the normal structure, including seminiferous tubules, interstitial tissue, Leydig cells, and germinal epithelium (Fig. 8A and B). In the AC-treated group, seminiferous tubules appeared degenerated. The germinal epithelium desquamated in some seminiferous tubules, leaving wide spaces around (Fig. 8C). The tubule lumen was devoid of spermatozoa, and the interstitial tissue and Leydig cells appeared vacuolated (Fig. 8D). A noticeable improvement in testicular tissue was observed in the AC + ADMSCs group (Fig. 8E and F). Most seminiferous tubules exhibited regular borders of basement membranes and a narrow lumen filled with spermatozoa. Leydig cells appeared normal in the vacuolated interstitial tissue.

**Fig. 9:**
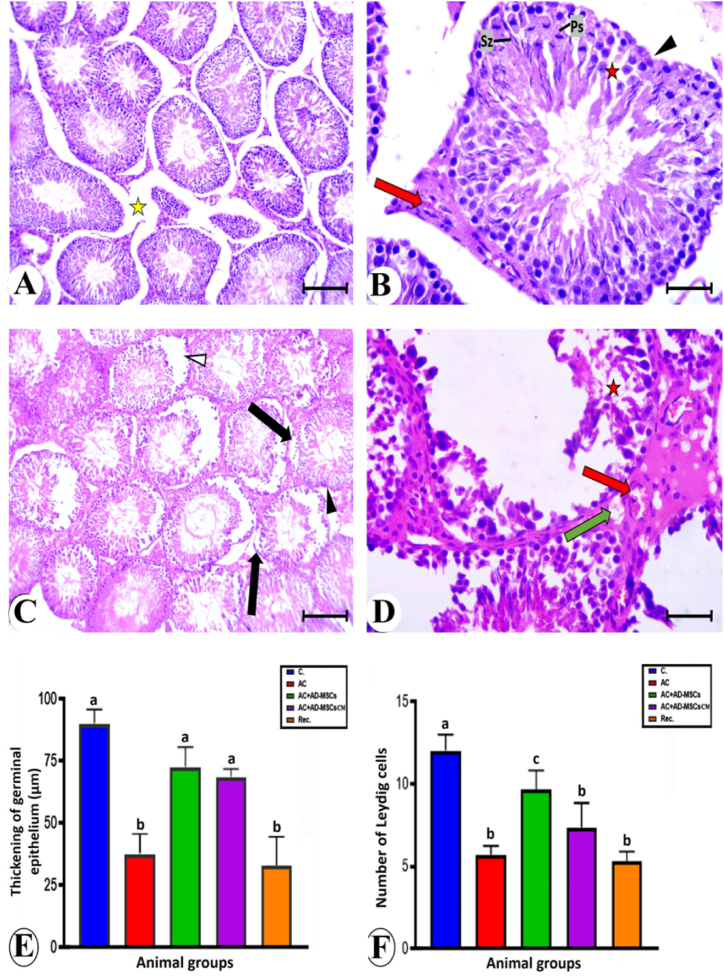
Photomicrographs of testis sections stained by H&E, bars = 100 μm (A and C) and 50 μm (B and d). **a and b:** AC + AD-MSCs CM group showing irregular border of seminiferous tubule (▲), germinal epithelium with prominent spermatocytes at different stages of division (Ps) and spermatozoa (Sz), spaces between germinal cells (red asterisk), congested blood vessel (red arrow) in the interstitial tissue, and edema (yellow asterisk). **C and D:** Rec. group showing irregular seminiferous tubule (▲), desquamation of the germinal epithelium to the lumen of the tubule (Δ), separation of germinal epithelium from its basement membrane (arrow), spaces between ill-defined germinal cells (red asterisk), vacuolation of interstitial tissue (green arrow), and congested blood vessel (red arrow). E and F: Thickening of the germinal epithelium (μm) and number of Leydig cells in the different experimental groups. Data are represented as the mean ± S.E. Unlike superscript letters in the same column are significantly different P < 0.05. or 0.001. (For interpretation of the references to color in this figure legend, the reader is referred to the Web version of this article.)

In the AC + ADMSCs-CM group, some seminiferous tubules appeared nearly normal, but others had disorganized germinal epithelium. Edema was detected in the interstitial spaces. The spermatozoa filled the lumen of the seminiferous tubules (Fig. 9A and B). Marked deterioration and degeneration of testicular tissue occurred in the recovery group. The seminiferous tubules were desquamated to the center germinal epithelium (Fig. 9C). The germinal epithelium cells with condensed nuclei, and the lumen of the tubules were devoted to spermatozoa. The interstitial tissue and Leydig cells showed marked vacuolation (Fig. 9 D). The histopathological lesions of the experimental groups are scored in Table 2. The germinal epithelium thickening and the number of Leydig cells were decreased in the AC and recovery groups versus that of the control group. They return nearly to a normal state when treated with AD-MSCs and AC + AD-MSCs-CM (Fig. 9E and F).

**Table (2):** The histopathological lesions in the examined groups.

Lesions	Groups				
	C	AC	AC+ AD-MSCs	AC+ AD-MSCs CM	Rec.
Edema	–	++	++	++	+++
Tubular atrophy	–	+++	+	++	+++
Disorganized germinal epithelium	–	+++	+	+	+++
Vacuolar degeneration in germinal epithelium	–	+++	+	++	+++
Undefined cells of germinal epithelium	–	+++	+	+	+++
Mitotic figures in primary spermatocytes	+++	–	++	+++	–
Seminiferous tubules lumen without spermatozoa	–	+++	+	+	+++
Vascular congestion	–	++	+	+	+++
Vascular dilatation	–	++	+	+	+++
Ledig cells degeneration	–	+++	+	++	+++

(-)Absent lesion, **(+)** Slight (<25 %), **(++)** Moderate (from 25 to 50 %), and **(+++)** Severe (>50 %).

### 3.9 Collagen fibers examination

**Fig. 10:**
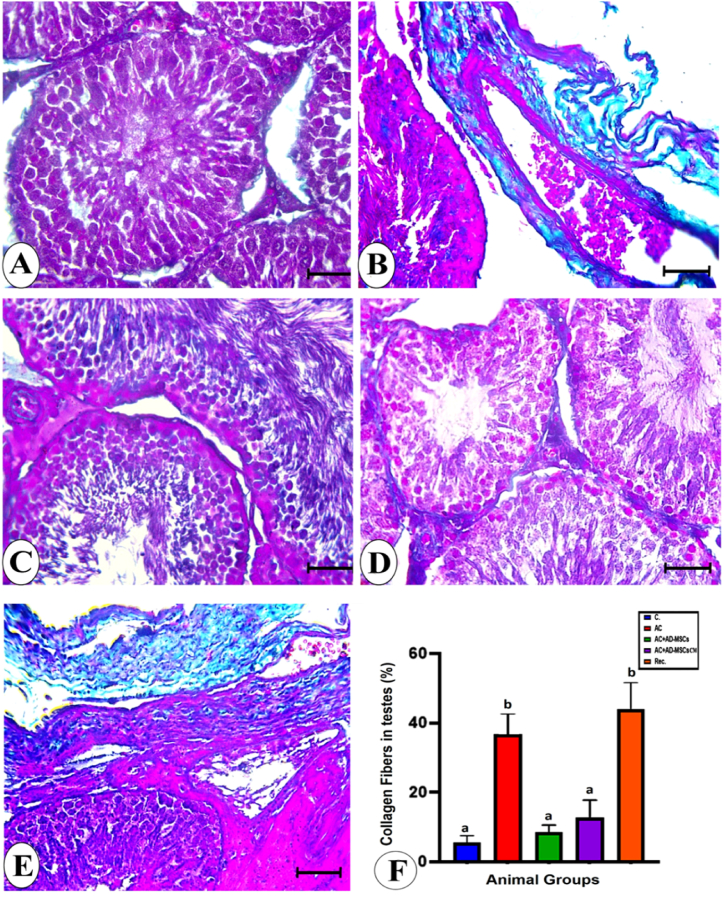
Photomicrographs of testis sections from experimental animal groups stained by Masson's trichrome, bar = 50 μm **A**: Control group showing mild amount of collagen fibers. **B:** AC showing marked increase of collagen fibers amount represented by green color. **C and D:** AC ± AD-MSCs and AC + AD-MSCs CM groups showing nearly normal amount of collagen fibers. **E:** Rec. group showing great amount of collagen fibers. **F:** Percentage of collagen fibers in testis sections from all experimental groups. Data are represented as the mean ± S.E. Unlike superscript letters in the same column are significantly different P < 0.001. (For interpretation of the references to color in this figure legend, the reader is referred to the Web version of this article.)

The collagen fibers in the control, AC, and recovery groups revealed tiny and excessive amount amounts of collagen fibers around the blood vessel and seminiferous tubules, respectively (Fig. 10 A, B, and E). Statistically, the collagen fibers in the AC and recovery groups were significantly increased versus that of the control group (Fig. 10F). In AC + AD-MSCs and AC + AD-MSCs-CM groups, the collagen fibers were significantly decreased compared to those of the AC group (Fig. 10C, D, and F).

## 4 Discussion

The present study suggests that AD-MSCs and AD-MSCs-CM have the potential to regenerate and repair the testes from the detrimental effects of acrylamide. That aligns with various reports highlighting the immunomodulatory, antioxidant, and anti-inflammatory activities attributed to MSCs [43,44]. Studies have demonstrated that AD-MSCs can reduce levels of apoptotic proteins and promote neuroectodermal differentiation, contributing to repair processes [30,45,30]. This study demonstrated the positive expression of CD90 and CD105 and the negative expression of CD45 and CD34 in the cultured isolated MSCs. Our findings align with these previous investigations [16,46].

Roasted, baked, or fried meals produce acrylamide; microwaved or boiled meals do not generally make acrylamide [47,48]. Our morphometric and immunohistochemical investigations revealed that the AC group's significantly elevated P53 activity in the testis was linked to various diseases, including severe testicular injury, poor spermatogenesis, and testis DNA fragmentation. P53 is a hub where many signaling pathways converge to carry out cell apoptosis. When it is activated, membrane proteins are destroyed, chromatin condensation occurs, and DNA breaks down [16,49].

The current study revealed that AC treatment and recovery led to the stimulation of oxidative stress and ROS. Indicators of ROS included elevated levels of lipid peroxidation (LPO) and DNA fragmentation in the testes. Excessive LPO levels induce apoptosis by activating pro-apoptotic pathways via P53. Numerous studies have highlighted AC-induced organ toxicity [3,4]. The reproductive toxic effects of AC have been investigated in laboratory animals across various concentrations and exposure times, resulting in outcomes such as decreased testicular weight, abnormal sperm, and reduced fertility rates [50,51]. Additionally, in rodent models, the detrimental effects of AC. on liver and renal tissues have been investigated [2,52].

DNA damage in cells triggers the accumulation of p53 in the nucleus and mitochondria, leading to the transcription of its target genes, such as mdm2, p21, bax, and puma. Subsequent outcomes include cell cycle arrest at the G1/S boundary and the initiation of cell death [53]. However, evidence suggests that AC ions may participate in a Fenton reaction, generating harmful oxygen radicals that can lead to DNA strand breakage [54].

Our findings revealed a significant increase in the mean values of DNA damage parameters. These results indicated that acrylamide exposure for 4 weeks, followed by a recovery period, induces elevated DNA damage in the testes tissues. These findings are consistent with the findings of **Arif et al.** [55], who found that lead produced DNA damage in erythrocytes and the spinal cords of rats. This damage was manifested as a significant rise in the mean values of the tail moment and olive tail.

Furthermore, previous studies have explained that increased nitric oxide (NO) from exposure to hazardous chemicals disrupts cellular respiration and induces apoptosis, contributing to DNA damage [20]. AC has been shown to alter the number and structure of chromosomes, induce micronuclei formation, break single and double strands of DNA, and form adducts with DNA. Following AC administration at various dosages to mice for 7 and 14 days, the tail moment significantly increased, indicating that AC-induced DNA damage is dose- and time-dependent [56,57,58].

The study demonstrated a significant reduction in DNA damage in the testes tissues following treatment with AD-MSCs and AD-MSCs-CM, as indicated by a decrease in the mean values of tail olive, tail length, tail moment, and comet length. Consistent with our results, El-shater et al. [21] reported a decrease in DNA damage to the spinal cord in MSC-treated rats exposed to lead Acetate using the comet assay technique. This improvement may be attributed to the anti-inflammatory and antioxidant activities of MSCs, leading to a decrease in free radical levels and an increase in antioxidant enzyme levels [59]. The amelioration that AD-MSCs provided may be attributed to its intrinsic antioxidant and free radical scavenging properties associated with its constituent bioactive components that repair DNA damage. MSCs secreted large amounts of trophic activity, and bioactive molecules such as growth factors and cytokines served a variety of functions, including inhibiting apoptosis and fibrosis and repairing DNA damage [21]. According to certain publications, injecting MSCs into atrophic seminiferous tubules may alleviate infertility and give functional evidence in favor of stem cell self-renewal and an increase in stem cell quantity during transplantation-induced regeneration [60].

Furthermore, AD-MSC-CM was shown to promote apoptosis and decrease HCC cell line proliferation, according to Dubey et al. [61] research. Extravascular vesicles (exosomes and microvesicles) and soluble mediators, known to have therapeutic effects in regenerative medicine, are secreted by CM-ASCs [62]. Furthermore, exosomes from human mesenchymal stem cells hESC-MSCs are equally effective against various forms of DNA damage [63]. According to our research, co-culturing AD-MSCs with their soluble mediator instead of utilizing their CM resulted in a more notable inhibition. Our hypothesis proposes that a mechanism involving cell-cell contact underlies this process. It suggests that interactions between AD-MSCs and testis cell receptors and ligands contribute to increased proliferation and inhibition of cell death [20]. Soluble mediators such as growth factors, cytokines, interleukins (IL-1Ra, IL-6, IL-7, IL-8, and IL-11), transforming growth factor-beta 1 (TGF-1), and granulocyte-colony stimulating factor (G-CSF) obtained from the conditioned medium (CM) of cultivated MSC; have been demonstrated in numerous studies to influence pro- and anti-inflammatory, pro-angiogenic, pro-apoptotic, and neurotrophic [25].

According to Yang et al. [64], the anti-tumor protein p53 affects the apoptotic pathway and may alter the levels of the Bcl-2 protein family. The immunoblotting results verified that AC-induced death of kidney and liver cells was caused by increased activation of p53, Bax, cytochrome *c*, and caspase-3, as well as decreased Bcl-2 protein expression [2].

The findings demonstrated a definite up-regulation of p53 (Tumor suppressor and pro-apoptotic marker gene) expression in the spermatogenic cells from the AC treatment group. AC led to oxidative stress and apoptosis in the rat testes employed in this investigation. Additionally, the spermatogenic cells' expression of p53 was reduced by AD-MSCs treatment [12,65].

Furthermore, after receiving AC treatment, an in vivo investigation on male rats revealed a noticeable decrease in spermatogenic cells in testicular injury, possibly related to germ cell death [66]. According to a prior study, apoptotic protein levels have been demonstrated to be lowered by AD-MSCs treatments on different tissues [30]. In the situations above, MSCs did not seem to differentiate into distinct cell types; instead, they used their release of growth factors, bioactive substances, and cytokines in large quantities to impact a therapeutic outcome [67]. These elements play a variety of functions, including limiting the area of damage, promoting angiogenesis, inhibiting apoptosis and fibrosis or scarring at the sites of injury, and ultimately promoting the proliferation of tissue-specific and tissue-intrinsic progenitors [60]. MSCs exhibited trophic and tissue-regeneration effects when modulated.

In this study, a significant increase in bright green fluorescence was observed when apoptosis was induced by AC toxicity. This observation may be elucidated by the likelihood that AO, fluorescing brightly green upon binding to DNA, is present in apoptotic cells [30]. According to earlier research, the apoptotic nucleus appeared smaller with increasing basophilia due to DNA condensation into a solid, shrunken mass in the spermatogenic cells [68]. After four weeks of exposure to AC, followed by a two-week recovery period, a significant decrease in free, total testosterone serum and LPO levels was observed. Comparable disruptions to male reproductive hormones due to AC exposure were previously documented by Yildrim et al. [69] and Shahrzad et al. [70]. The decline in testosterone induced by AC exposure may be attributed to Leydig cell weakening, triggered by elevated LH secretion. The inverse correlation between estradiol and testosterone could stem from heightened aromatase production in adipose tissue, leading to the conversion of testosterone to estradiol.

The reports [71] clarified that lipid peroxidation in organisms results in MDA and exhibits cytotoxicity, causing proteins and DNA strand breakage. Oxidative damage can occur when hydroxyl radicals and superoxide anions react directly with DNA molecules, and the amount of damage inflicted by free radicals is indirectly reflected in the MDA level [58]. This explains why our findings point to oxidative stress as the primary mechanism for AC-induced damage in the testis.

Surprisingly, our investigations revealed the restoration testosterone, MDA, and apoptosis levels to normal ranges in the AC + AD-MSCs and AC + AD-MSCs-CM groups. This further corroborated the "homing theory," which postulates that stem cells detect injury sites, migrate to them, and then develop into cells specific to each organ, promoting organ homeostasis and tissue repair [58]. Relevant publications suggest that injecting MSCs into atrophic seminiferous tubules may provide functional evidence for stem cell self-renewal, and immunomodulatory functions [58]. The therapeutic role of AD-MSCs is also supported by their presence in the damaged seminiferous tubules and tubule lumen as shown in our results had migrated to the wounded testis. Notably, ferritin and transferrin receptor 1 genes have been identified as potential reporter genes for tracking the location and health of transplanted MSCs. The ability of these proteins to accumulate iron further supports their utility in detecting MSCs [47,72].

In our studies, AC exposure decreased spermatogenesis, abnormalities, increased collagen fiber deposition, and the enlargement of intercellular spaces all agree with earlier research [73]. ROS levels were associated with the down-regulation of cadherins crucial for germ and Sertoli cells' adhesion, facilitating their interaction with tight junction proteins in the blood-testis barrier [74]. It has been suggested that AC could potentially cause aberrant hepatic gene rise in COL1A1 mRNA expression brought on by AC expression, leading to the build-up of collagen, a significant clinical characteristic in numerous liver illnesses [75]. The current study mitigated histological alterations induced by AC after rats injection by MSC. Other researchers [76,77] found that a conditioned medium increased kidney proliferation and alleviated the detrimental effects of gentamicin. Sobh et al. [28] demonstrated that MSCs and their conditioned medium reduced lessened pathological alterations in acute renal injury induced by cisplatin. However, Xing et al. [78] and Salem et al. [29] discovered that MSCs, rather than conditioned medium, facilitated kidney healing following ischemia-reperfusion injury. Our finding supports that MSCs may have contributed to the re-establishment of spermatogenesis in two ways: either by differentiating into sperm or by maintaining the spermatogonial stem cells.

## 5 Conclusion

In conclusion, oral administration of acrylamide induces testis damage characterized by elevated P53 and LPO levels, DNA damage, and a significant increase in apoptosis. Acrylamide leads to substantial histopathological changes, fibrosis, and a reduction in both total and free testosterone levels. The therapeutic potential of stem cells, particularly AD-MSC transplantation, mitigates the adverse effects of acrylamide in testis more effectively than AD-MSC-CM.

## CRediT authorship contribution statement

**Mona M. Atia:** Writing – review & editing, Writing – original draft, Visualization, Validation, Supervision, Software, Resources, Project administration, Methodology, Investigation, Formal analysis, Data curation. **Aya Ahmed Badr EL-Deen:** Writing – original draft, Resources, Methodology, Formal analysis, Conceptualization. **Hanem.S. Abdel-Tawab:** Writing – review & editing, Visualization, Validation, Supervision, Conceptualization. **Alshaimaa.A.I. Alghriany:** Writing – review & editing, Writing – original draft, Visualization, Validation, Supervision, Resources, Project administration, Methodology, Formal analysis, Conceptualization.

## Ethical approval

We confirm that our study was conducted following ARRIVE guidelines.

## Data availability statement

Data will be made available on request.

## Funding

No.

## Declaration of Competing Interest

The authors declare the following financial interests/personal relationships which may be considered as potential competing interests: Mona M, Atia reports administrative support was provided by Assiut University. Mona M. Atia reports a relationship with Assiut University that includes: non-financial support. If there are other authors, they declare that they have no known competing financial interests or personal relationships that could have appeared to influence the work reported in this paper.
